# “ACT YOUR AGE!” YOUNG ADULTS’ CONCERN REGARDING PRESCRIPTIVE AGE STEREOTYPE VIOLATION

**DOI:** 10.1093/geroni/igae098.3250

**Published:** 2024-12-31

**Authors:** Amy Gourley, Alison Chasteen

**Affiliations:** University of Toronto, Toronto, Ontario, Canada; University of Toronto, Toronto, Ontario, Canada

## Abstract

A younger subjective or ‘felt’ age is often associated with positive health outcomes among adults over 60. However, a dearth of research examines young adults’ attitudes towards older people who report feeling ‘young at heart’. The degree to which a young adult feels concerned about violations of prescriptive age stereotypes (i.e., that older people should act their age) may predict their reactions to older adults who feel young at heart. The present work examines young adults’ (N = 296) attitudes toward older vignette characters described as feeling either their age (65), 45, or 25. Participants evaluated one of six possible characters and completed a measure of their concern that the character would violate prescriptive age stereotypes (e.g., “I am concerned that older people like the person described in the vignette will come to places where I like to hang out.”). Participants were more concerned that characters who felt 25 or 45 would violate prescriptive age stereotypes compared to characters who felt 65. Moreover, concern about characters’ potential stereotype violation predicted participants’ attitudes toward the character. Specifically, the more concerned participants felt, the lower they rated characters’ competence, particularly when the character felt 25 or 65. These findings suggest that older adults’ felt ages and younger adults’ concerns about preserving strict age-group boundaries may be key predictors of evaluations of older people. To develop interventions to reduce ageism, more work must consider younger people’s reactions to older adults’ felt ages as well as their concern regarding prescriptive age stereotypes.

